# Models for midwifery care: A mapping review

**DOI:** 10.18332/ejm/124110

**Published:** 2020-07-30

**Authors:** Tine S. Eri, Marie Berg, Bente Dahl, Helga Gottfreðsdóttir, Eva Sommerseth, Christina Prinds

**Affiliations:** 1 Faculty of Health Sciences, Oslo Metropolitan University, Oslo, Norway; 2 Sahlgrenska Academy, Institute of Health and Care Sciences, University of Gothenburg, Gothenburg, Sweden; 3 The Obstretic Unit, Sahlgrenska University Hospital, Gothenburg, Sweden; 4 Centre for Women’s, Family and Child Health, Faculty of Health and Social Sciences, University of South-Eastern Norway, Kongsberg, Norway; 5 Department of Midwifery, Faculty of Nursing, University of Iceland, Reykjavík, Iceland; 6 Women´s Clinic, Landspitali University Hospital, Reykjavík, Iceland; 7 Institute of Clinical Research, University of Southern Denmark, Odense C, Denmark; 8 Department of Research, University College South Denmark, Haderslev, Denmark

**Keywords:** midwifery, salutogenesis, philosophy, maternity care, models for care, woman-centeredness

## Abstract

**INTRODUCTION:**

According to WHO, midwives are found competent to provide evidencebased and normalcy-facilitating maternity care. Models for midwifery care exist, but seem to be lacking explicit epistemological status, mainly focusing on the practical and organizational level of care delivery. To make the values and attitudes of care visible, it is important to implement care models with explicit epistemological status. The aim of this paper is to identify and gain an overview of publications of theoretical models for midwifery care.

**METHODS:**

A mapping review was conducted with systematic searches in nine databases for studies describing a theoretical model or theory for midwifery care that either did or was intended to impact clinical practice. Eligibility criteria were refined during the selection process.

**RESULTS:**

Six models from six papers originating from different parts of the world were included in the study. The included models were developed using different methodologies and had different philosophical underpinnings and complexity gradients. Some characteristics were common, the most distinctive being the emphasis of the midwife–woman relationship, secondly the focus on woman-centeredness, and thirdly the salutogenic focus in care.

**CONCLUSIONS:**

Overall, scarcity exists regarding theoretical models for midwifery care with explicit epistemological status. Further research is needed in order to develop generic theoretical models with an epistemological status to serve as a knowledge base for midwifery healthcare.

## INTRODUCTION

All healthcare is based on values and attitudes that are sometimes explicitly expressed in theoretical frameworks or condensed as models for care, but which are mostly tacit. Such frameworks increase the facilitation of awareness of having an epistemological basis for healthcare, and function as important guiding tools for the organization of such healthcare. Maternity healthcare, including the period before, during and after childbirth and the parenthood transition, is subject to different epistemological statuses representing different professional and scientific traditions, including both midwifery and medical models^1-4^.

The approaches to health and illness affect the way in which care models are positioned. Models for care have emerged, often relating to epistemological status, as well as being appealing at the practical level in terms of how to organize care. However, entangling the practical and epistemological levels of a model may require a more analytical approach, since these two levels often overlap, thus leaving merely one lens through which we are to understand health and hence organize care around. Opposed models of care exist in the field of maternity care, and especially around childbirth, which have been labelled for example ‘medical’ versus ‘social and women-centered’^5^, ‘technocratic’ versus ‘holistic’^6^, or ‘pathological’ versus ‘salutogenic’ models^7^. The reason for these opposed perspectives should be sought in relation to the choice of positioning pregnancy and childbirth in the medical specialty of obstetrics: *‘Pregnancy in western society, in fact, straddles the boundary between illness and health: the status “pregnant” is unclear in this regard and women perceive that others are not sure whether to treat them as ill or well’*^8^.

Whether pregnant women are regarded as ill or well; pregnancy, childbirth and the surrounding maternity services are culturally sensitive. This leaves women, their partners and children in various culturally dependent statuses at the global level. It also leaves maternity care in the hands of different health professionals. Facilitating health in childbirth, however, is a complex task that successively includes a risk management perspective, driven by rules and protocols that overlook individual needs and circumstances^9^. The situation of one pregnant woman is influenced by factors far beyond her needs and circumstances, and practitioners and researchers have thus put forth theories intended to shed light on the complexity of healthcare systems like maternity care^10,11^. Furthermore, a taxonomy for complexity theory has been developed to increase understanding of how some techniques become widely adopted although on a country-specific basis^12,13^.

To care for a normal physiological pregnancy and childbirth and secure normalcy, professional midwives seem to be the relevant choice^14^. Unfortunately, professional midwives are only available in certain parts of the world, whereas in other parts, childbirth attendants are primarily obstetricians, obstetric nurses or practically trained laymidwives^15^. Models for care with explicit epistemological status are therefore important in order to implement evidence-based and normalcy-facilitating care^16^. Several care models already exist, but a previous mapping review that explored the characteristics of antenatal care models found that several models lacked an explicit epistemological basis^17^. Some researchers have developed and attempted to implement different models for care^18-21^. Furthermore, the International Confederation of Midwives (ICM) has developed a core document that outlines the organization’s model of midwifery care with an underpinning philosophy of care^22^.

There is no consensus about what is meant by a model, and after reviewing the literature, the distinction between a care delivery model outlining practical details about care provision and a theoretically-developed care model with a clear epistemological basis seems blurry. According to Walker and Avant^23^, the graphic representation of a theoretical framework can be called a model, hence the term ‘theoretical model’ in order to make the distinction from organizational models of care. However, there appears to be a gap in the overview of existing models and, to our knowledge, no overview of existing scientifically-developed theoretical models for midwifery care has been published. The aim of this paper is therefore to identify and gain an overview of publications containing theoretical models for midwifery care.

## METHODS

To fulfil the objective of the paper, we conducted a mapping review, which is a method designed to provide a wide overview of a research area, establish if research evidence exists on a topic and provide an indication of the quantity of the evidence. The method is used to map out and categorize existing literature on a particular topic and identify gaps in research literature from which to commission further reviews and primary research^24,25^. According to the SALSA framework, the main types of literature reviews are classified into four key stages: 1) Search, 2) AppraisaL, 3) Synthesis, and 4) Analysis. For a mapping review, the search for literature is extensive and systematic^26^. Usually, there is no appraisal or formal quality assessment as the aim is limited to mapping out and categorizing existing literature. The synthesis stage of the mapping review focuses on the visualization of data, which may be graphical and tabular. The analysis stage often involves characterizing quantity and quality and other key features of relevance to the review questions^24,26^. A description of how we applied these stages to our review is now given.

### The search phase

This phase comprised an extensive, systematic search in relevant databases and a systematic screening and selection of studies.

#### Eligibility criteria

Inclusion and exclusion criteria were established in advance, and subsequently further developed along with the screening process. We did not pose any time limit on the searches. Inclusion criteria were as follows, and all criteria had to be fulfilled:

- Full text available, papers published in peer-reviewed journals, studies that describe a theoretical model or theory for midwifery care (or some part of a model or theory), studies that describe a model or theory that either have or are intended to impact clinical practice.

Exclusion criteria were as follows, and one criterion was enough for exclusion:

- Studies that describe models that are strictly philosophical (which are not intended to impact practice), studies that describe organizational models only (care provision, service models, care delivery etc.) without describing or explaining in part or in whole the theoretical model or theory of midwifery care underpinning the proposed organization of care, and studies that describe the practical details of implementing care without giving the underlying concepts.

#### Search strategy

The search strategy was designed and developed with the assistance of a specialist librarian. A scoping search including the keywords midwife/midwifery, model/theory/framework, nursing models/nursing theory and woman-centered care was conducted in the MEDLINE, Cinahl, and Maternity and Infant Care databases in May 2018. We continued by refining the study objectives, choice of keywords and inclusion/exclusion criteria before conducting a systematic search in September 2018. The following databases were included: Ovid MEDLINE(R), Ovid Nursing, PsycINFO, Cinahl, Trials (Cochrane Library), Maternity and Infant Care, Academic, Scopus, and Web of Science. Keywords included a variety of terms used to describe midwifery models and care. Language was limited to include papers in English, Danish, French, German, Icelandic and Norwegian.

In total, 11132 citations were identified. The search results were imported into a reference manager software (EndNote) and duplicates were removed, leaving 5449 titles. When imported into a systematic review management software, a further 55 duplicates were removed, leaving 5394 titles and abstracts to be screened for inclusion.

### Selection and screening of studies

We managed the screening process in the review management software Covidence, and we distributed the titles and abstracts randomly among the review team comprising the six authors. The screening and selection process consisted of two subsequent phases. The first was the title and abstract screening, where 5159 studies were found to be irrelevant to the aim of the review in accordance with the inclusion and exclusion criteria. The second phase then encompassed 234 papers for further investigation. These were randomly distributed among the review team, and two reviewers assessed each paper for inclusion to obtain consensus.

During this process, it was necessary to discuss and refine the eligibility criteria because the term ‘model’ was used in different ways and had different meanings in the sample. It was necessary to specify that we were not looking for studies that describe organizational models only, or studies that describe the practical details of implementing care; the aim of the review was to identify theoretical models for midwifery care. We resolved conflicts either by assigning a third reviewer, or by discussing them in the team. An example is the extensive discussion involving the whole team about the assessment and possible inclusion of two important papers: The Lancet paper on the QMNC framework^27^ and the Cochrane review on midwife-led continuity models versus other models of care^19^. Neither of the two papers were included in the final selection. We excluded both because they do not describe the development of a theoretical model for midwifery care. The former^27^ describes a framework on the macro-level about how to secure quality maternity and newborn care in all settings. The latter^19^ compares outcomes of different ways of organizing maternity care.

**Figure 1:**
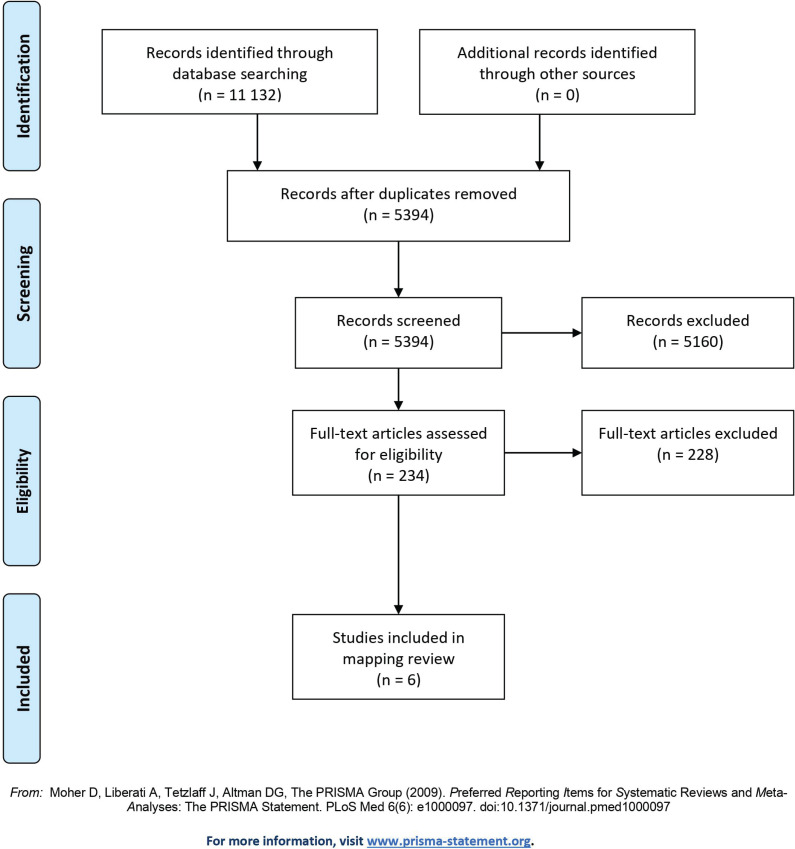
Flow diagram

Of the 234 papers assessed in full text, further discussions on inclusion led to the selection of 10 papers for more detailed review. These were discussed in relation to the inclusion criteria and of the ten, four were found to be outside these criteria. The flow of the selection of studies is shown in Figure 1.

### The analysis and synthesis phases

We extracted the following data from each paper: authors and year of publication, setting for the study, name of the model, aim of the model, methodology behind the development of the model, philosophical ideas underpinning the model, description of the concepts on which the model is built, scope of the model, and suggested use of the model. Finally, we noted if there was an illustration of the model.

The described models were analyzed in terms of complexity and orientation. According to Kannampallil^28^, the range of complexity depends on the number of components and their interrelatedness. Interrelatedness refers to the influence of system components on each other. We placed the models on a continuum, stretching from very simple to very complex. The orientation of the models was mainly towards care, relationships, professionalism or health.

Furthermore, we mapped the components of each model to identify similarities and differences between them. Inspired by the ‘idiomatic translation’ of metaethnography^29^, we chose one reference paper as the vantage point. We worked with each model and separated its components to see if it could be understood in the same way as the components of the reference paper, or that the authors used different concepts to describe identical meaning content. If not, we added a new line for each new concept that was not covered by the previous models in the map.

## RESULTS

The findings are presented in three sections consisting of an overview, followed by a brief description of each model and concluding with a mapping of relevant components of the models. In the following presentation, each model is given a short label based on the original paper.

### Overview

The six included publications describe six models (labels in brackets):

1. Women-with-midwives: a model of interdependence (Women-with-midwives)^30^
2. A model of exemplary midwifery practice (Exemplary midwifery practice)^31^
3. A midwifery model of care for childbearing women at high risk: genuine caring in caring for the genuine (Midwifery at high risk)^32^
4. A woman-centred childbirth model (Woman-centred SA)^33^
5. The primacy of the good midwife in midwifery services: an evolving theory of professionalism in midwifery (The primacy of the good midwife)^34^
6. A midwifery model of woman-centred childbirth care – In Swedish and Icelandic settings (Woman-centred Nordic)^35^

Data for the developed models were collected in New Zealand and Scotland (Women-with-midwives)^30^, the United States (Exemplary midwifery practice)^31^, Sweden (Midwifery at high risk)^32^, South Africa (Woman-centred SA)^33^, and Sweden and Iceland (The primacy of the good midwife)^34^ (Woman-centred Nordic)^35^. An overview of descriptive data for the models is given in Table 1.

**Table 1:** Descriptive data of the included models

*Name of the model*	*Authors (year)*	*Setting/Country*	*Aim and scope of the model*	*Method for development of the model and data sources*	*Philosophical ideas or professional authoritative knowledge underpinning the model*
Women-with-midwives-with-women: a model of interdependence	Fleming^30^ (1998)	New Zealand and Scotland	Aim: to develop a research-based conceptual model of midwifery practice Scope: pregnancy, labour and postnatal period	Grounded Theory design. Data collection: unstructured interviews with midwives, women, and observations of midwife-client interactions. Participants: 250 midwives and 219 clients. Primary data sources	Rejection of the medical model of childbirth
A model of exemplary practice: Results of a delphi study	Kennedy^31^ (2000)	USA	Aim: to describe exemplary midwifery practice Scope: pregnancy, birth and post-partum period	Delphi method including a sample of 52 midwives and 61 recipients of care. Primary data sources	Critical and feminist theories
A midwifery model of care for childbearing women at high risk: genuine caring in caring for the genuine	Berg^32^ (2005)	Sweden	Aim: to describe the essence of the midwifery model of care for women at high risk during childbearing Scope: midwifery care for pregnant women at high risk	A research synthesis of three qualitative interview studies was performed, of which two presented women’s experiences with complicated childbirth, and one reported midwives’ experiences of caring for women of high risk. Secondary data sources	Childbearing as a normal process
A woman-centred childbirth model	Maputle^33^ (2010)	South Africa	Aim: to develop a ‘woman centred’ childbirth model that could be used to assist the attending midwives in the facilitation of mutual participation while managing mothers during childbirth Scope: childbirth	A qualitative design with interviews, participant observation and unstructured conversations of 24 mothers and 12 attending midwives within 24 hours of the delivery. Primary data sources	Empowerment and egalitarism
The primacy of the good midwifery services: an evolving theory of professionalism in midwifery	Halldorsdottir & Karlsdottir^34^ (2011)	Iceland	Aim: to construct a theory on the empowerment of women in the childbearing process with emphasis on the midwife´s professionalism Scope: the childbearing period	Theory synthesis according to Walker and Avant (2004) comprising three steps. Nine studies of experiences of women as clients of midwifery and health care formed the basis of the work. Secondary data sources	Not clear
A midwifery model of women-centred childbirth care - in Swedish and Icelandic settings	Berg et al.^35^ (2012)	Sweden and Iceland	Aim: to define and develop an evidence-based midwifery model of woman centred care in Sweden and Iceland Scope: care during labour and birth	Findings from 12 previously published studies were used in a qualitative hermeneutic design, eight based on interviews with women and four presenting interviews with midwives. Secondary data sources	Childbirth is viewed as a normal social event taking place within the family

Methods used to develop the models were; grounded theory (Women-with-midwives)^30^, the Delphi method (Exemplary midwifery practice)^31^, research synthesis (Midwifery at high risk)^32^, qualitative design (Woman-centred SA)^33^, theory synthesis (The primacy of the good midwife)^34^, and qualitative hermeneutic design (Woman-centred Nordic)^35^. Three of the models were based on original empirical data (Women-with-midwives)^30^, (Exemplary midwifery practice)^31^, (Woman-centred SA)^33^, two were secondary analyses of original studies (Midwifery at high risk)^32^, (The primacy of the good midwife)^34^, and one model was developed through secondary analysis of original studies followed by validation testing (Woman-centred Nordic)^35^.

**Table 2:** Characteristics of included models

*Name of the model*	*Main concepts building the model*	*Orientation of the model*	*Suggested use of the model*
Women-with-midwives-with-women: a model of interdependence (Fleming 1998)^30^	Six major categories are presented in three pairs representing respectively women’s and midwives’ place in the model: Attending and presencing Supplementing and complementing Reflection and reflexivity Together these categories form the essence ‘reciprocity’	Relationship-oriented	The model offers the beginning of documentary evidence of the essence of the midwife-client relationship: it has potential applicability for both midwifery education ad practice
A model of exemplary practice: results of a delphi study (Kennedy 2000)^31^	The model comprises three dimensions with associated outcomes: *Therapeutics* Outcome: Optimal health of the woman and/or infant in the given situation *Caring* Outcome: The woman and family have a health care or birth experience that is respectful and empowering *Profession of Midwifery* Outcome: Enhancement of the profession of midwifery	Health-oriented, Profession-oriented	The model provides a structure for future research
A midwifery model of care for childbearing women at high risk: genuine caring in caring for the genuine (Berg 2005)^32^	The general structure consists of three constituents, each constituent comprises several elements: *A dignity-protective relationship* Mutuality/trust/an ongoing dialogue/shared responsibility/enduring presence *Embodied knowledge* Genuineness towards oneself/theoretical knowledge/practical knowledge/intuitive knowledge/reflective knowledge. *A balancing of the natural and medical perspectives* Supporting normalcy/exhibiting sensitivity for the genuine	Care-oriented	The development of this model of midwifery care for childbearing women at high risk can serve as a prototype for a similar development of a model of education for both high- and low-risk pregnant women
A woman-centred childbirth model (Maputle 2010)^33^	The process of providing woman-centred care during childbirth would take place in three phases: • Phase 1 (dependence phase) describes differences and similarities in the encounters • Phase 2 (interdependence phase) describes strategies to enhance facilitation • Phase 3 (independence phase) describes outcome that strives to achieve respectful relationships, equality, power sharing and responsibility, partnership, information & decision-making and dialogue	Relationship-oriented	The model is aimed at enhancing the provision of woman-centred care which will facilitate mutual participation and responsibility-sharing, creation of opportunities for information sharing and empowering, open communication and listening, accommodative midwifery actions and maximising of human and material infrastructure during childbirth
The primacy of the good midwifery services: an evolving theory of professionalism in midwifery (Halldorsdottir & Karlsdottir 2011)^34^	The professionalism of the good midwife is constructed from five mains aspects: The midwife’s professional caring The midwife’s professional wisdom The midwife’s development The midwife’s interpersonal competence The midwife’s professional competence	Profession-oriented	The theory has implications for midwifery education and practice
A midwifery model of woman-centred childbirth care childbirth care – in Swedish and Icelandic settings (Berg et al. 2012)^35^	The main components are three central and two surrounding themes: Central themes: *A reciprocal relationship* Presence/affirmation/ availability/ participation *A birthing atmosphere* Calm/trust/safety/strengthening/supporting normalcy *Grounded knowledge* Different kind of knowledge/embodied knowledge/knowledge in relation to woman Surrounding themes: Cultural context with promoting and hindering norms and balancing act in facilitating woman-centred care	Care-oriented	The model could be applied to midwifery care in general throughout pregnancy, birth and postpartum care. The model can be used as a guide for everyday midwifery practice. The model could serve as a broad theoretical framework for midwifery practice, education, management and research

**Figure 2:**
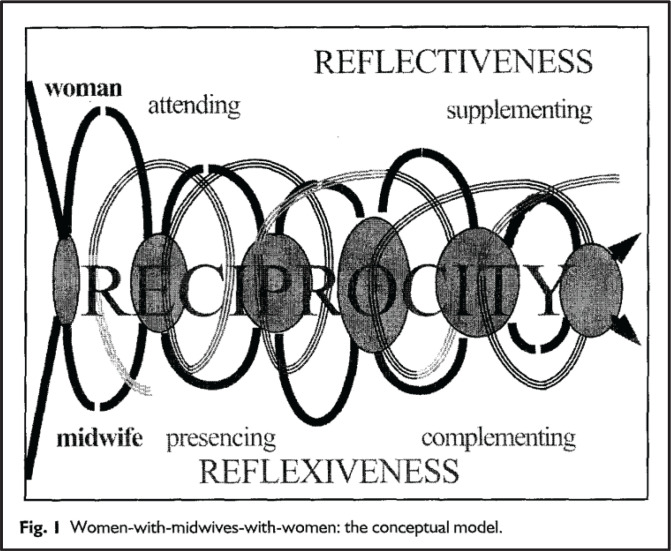

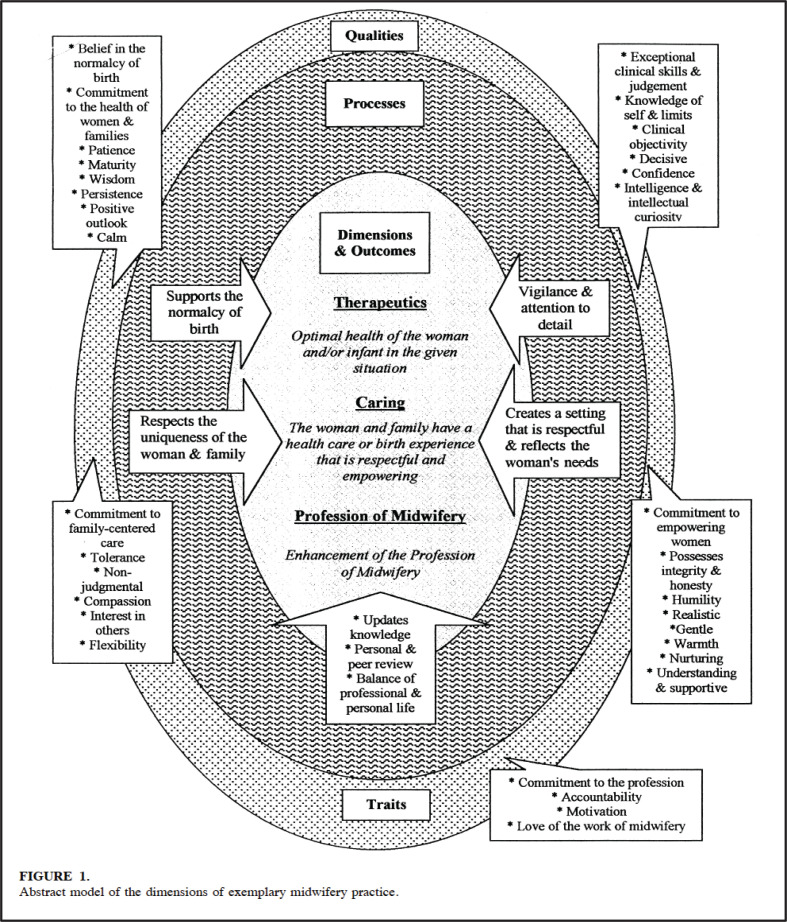
Overview over two included visual models (Fleming, 1998 and Kennedy, 2000) (with permission from the publishers)

**Figure 3:**
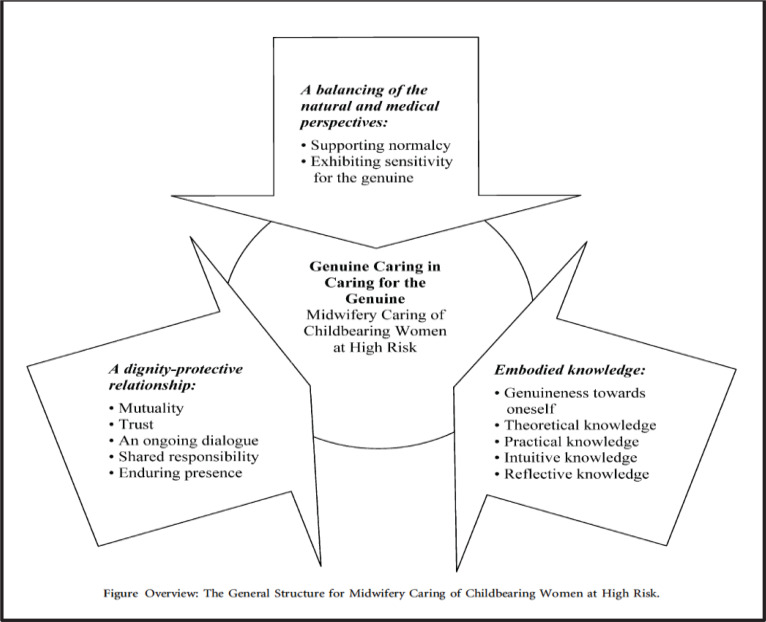

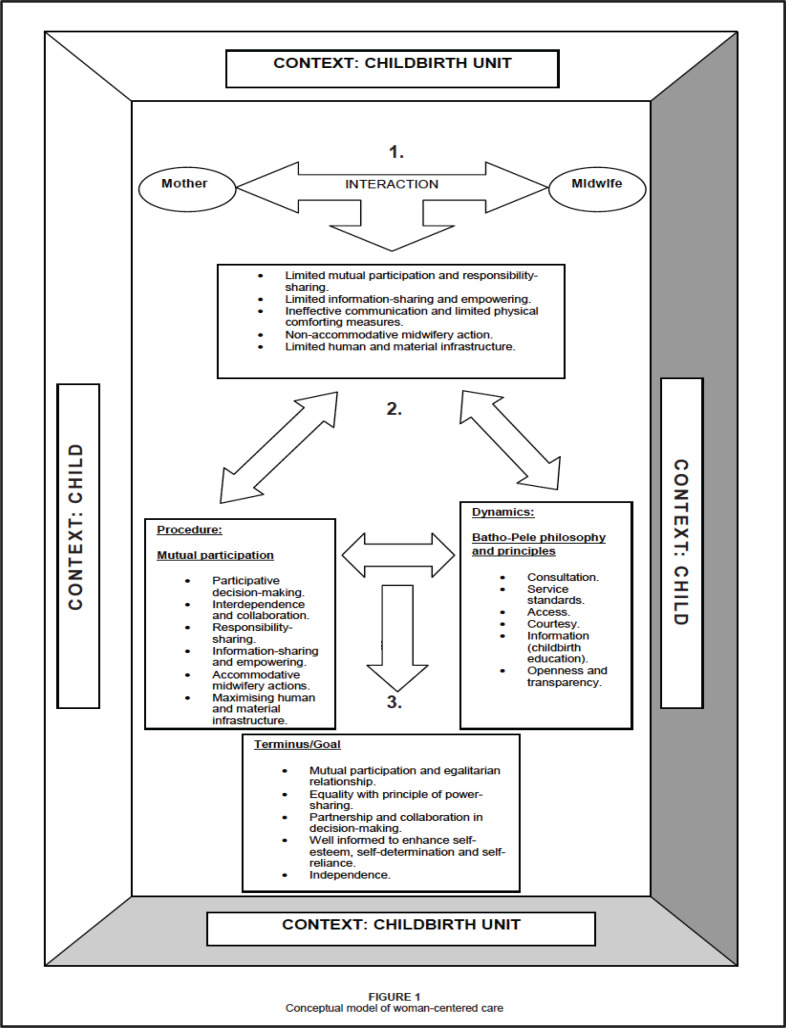
Overview over two included visual models (Berg, 2005 and Maputle, 2010) (with permission from the publishers)

**Figure 4:**
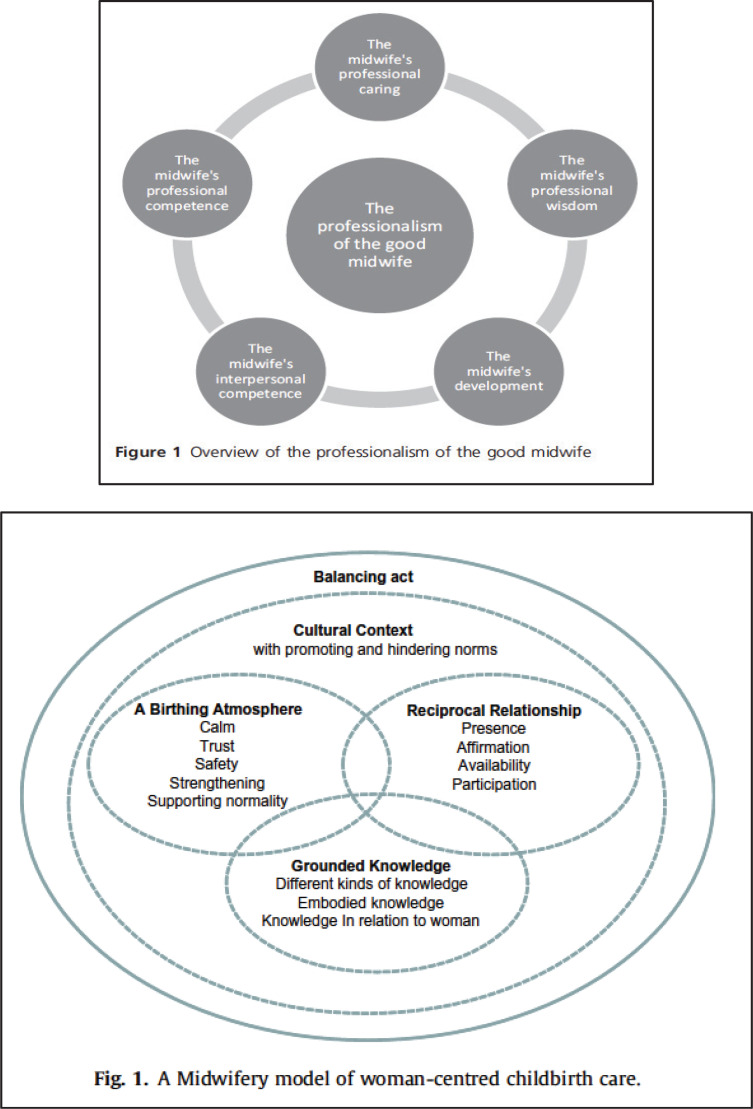
Overview over two included visual models (Halldorsdottir & Karlsdóttir, 2011 and Berg et al, 2012) (with permission from the publishers)

In terms of assessed complexity on a continuum ranging from very simple to very complex, one of the models was perceived as very simple (The primacy of the good midwife)^34^ and two as very complex (Exemplary midwifery practice)^31^, (Woman-centred SA)^33^. The three remaining models (Women-with-midwives)^30^, (Midwifery at high risk)^32^, (Woman-centred Nordic)^35^ were placed somewhere in the middle of the continuum. An overview of the characteristics of the models is given in Table 2, and a visualization of the models in Figures 2–4.

#### Brief description of the six models

##### Model 1: ‘Women-with-midwives’

A model of interdependence that is oriented towards the woman–midwife relationship. It was developed on the basis of unstructured interviews and observations of interactions between midwife and client, collected in New Zealand and Scotland, using a grounded theory approach. The model consists of six major categories, formed as three pairs, representing women and midwives, respectively: ‘attending – presencing’, ’supplementing – complementing’, and ‘reflection – reflexivity’. The model represents a relationship that is episodic and not always equally balanced, and the basic social process of reciprocity embraces the whole midwife–client relationship. In the visualization of the model, it is shown how the midwife and client meet as strangers, have a period of meetings that can be episodic and not always balanced, and that there are contextual factors influencing the relationship^30^ (Figure 2).

##### Model 2: ‘Exemplary midwifery practice’

The model was developed based on a framework of three aspects, and with an orientation towards health for woman and families, and towards midwifery professionalism. A Delphi study was conducted in the US with a sample comprising exemplary midwives and women who had received their care. The model encompasses essential alignments within three dimensions. The outcome of the first dimension, ‘therapeutics’, is that the woman and/or infant in the given situation has optimal health. The outcome of the second dimension, ‘caring’, is that the woman and the family have a respectful and empowering healthcare and birth experience, while the outcome of the third dimension, ‘profession of midwifery’, is that the profession of midwifery is enhanced. These three dimensions and outcomes are placed in a circle in the center on a background of midwives’ qualities and traits^31^ (Figure 2).

##### Model 3: ‘Midwifery at high risk’

This is a practice care-oriented model, which describes what constitutes ideal midwifery care for childbearing women at high risk. It was developed through a research synthesis of three phenomenological interview studies in Sweden with women (n=2) and midwives (n=1), in which the author served as the primary investigator. The essence of the model is ‘genuine caring in caring for the genuine’, which includes three constituents: ‘a dignity-protective relationship’, ‘embodied knowledge’, and ‘a balancing act of the natural and medical perspective’. Each constituent comprises two to five elements^32^ (Figure 3).

##### Model 4: ‘Woman-centred SA’

The model was developed to assist midwives in the facilitation of mutual participation during childbirth and through enhancing the implementation of the ‘Batho–Pele principles’ for consultation, service standards, assessment, courtesy, information, openness and transparency. A qualitative design was used. Data were collected from two interview and observation studies with women having given birth at one hospital in South Africa and with their attending midwives. The model is complex and strongly orientated towards relationships. The process of providing woman-centred care takes place in three phases: 1) the ‘dependence phase’ signified by limited mutual participation between the mother and the midwife; 2) the ‘interdependence phase’ including strategies to facilitate mutual participation and comprising procedures and dynamics; and 3) the ‘independence phase’,

which focuses on outcomes of care. There is a dynamic relationship between the phases, which exists in the context of the childbirth unit and the child^33^ (Figure 3).

##### Model 5: ‘The primacy of the good midwife’

In this model the midwife’s professionalism is central. Through using a theory synthesis method, data were analyzed from 9 studies conducted by any one or more of the authors, sometimes in collaboration with other researchers. Most of the original data were collected in Iceland, except in one study that was a secondary analysis of studies conducted in Iceland, Sweden and Finland. The professionalism of being a good midwife is constructed from five main aspects: ‘the midwife's professional caring’, ‘the midwife's professional competence’, ‘the midwife's interpersonal competence’, ‘the midwife's development’, and ‘the midwife's professional wisdom’^34^ (Figure 4).

##### Model 6: ‘Woman-centred Nordic’

This model is oriented towards practical midwifery care during labor and birth. It was developed through a synthesis of 12 interview studies with women (n=8) and midwives (n=4) focusing on their experiences of childbirth. The studies were conducted by one or more of the three authors, mostly in collaboration with other researchers. The model consists of five intertwined themes. Three of these themes are central and overlapping: ‘reciprocal relationship’, ‘a birthing atmosphere’, and ‘grounded knowledge’. These are surrounded by two themes: ‘cultural context’ and ‘the balancing act’, which describe how care takes place in a cultural context comprising both promoting and hindering norms, and how midwives then need to conduct a balancing act in striving towards woman-centred care^35^ (Figure 4).

#### Mapping the components of the models

We chose the most recently published model, ‘the midwifery model of woman-centred childbirth care’ (Women-centred SW)^35^, as the reference model when we mapped the central components of each model to identify similarities and differences. We mapped the remaining models with their respective central concepts in relation to this reference model. The names given to the components used to describe the constructed models vary, the reason being that different qualitative methods have been used in the analyses, and because some models were deductively developed from already defined frameworks. Furthermore, the number of components varied between the models, from having components in only one line (Women-with-midwives)^30^ to six lines (Exemplary midwifery practice)^31^. Table 3 provides a matrix overview of the mapping of the components of each model.

**Table 3:** Mapping of the single components of the models

*A midwifery model of woman-centred childbirth care*	*Genuine caring in caring for the genuine*	*Women-with-midwives-with-women: a model of interdependence^a^*
Birthing atmosphere o Calm o Trust o Safety o Strengthening o Supporting normalcy	Supporting normalcy	
Reciprocal relationship o Presence o Affirmation o Availability o Participation	A dignity-protective relationship o Mutuality o Trust o An ongoing dialogue o Shared responsibility o Enduring presence	Reciprocity o Attending/Presencing o Supplementing/complementing o Reflectiveness/reflexiveness
Grounded knowledge o Different kinds of knowledge o Embodied knowledge o Knowledge in relation to woman	Embodied knowledge o Genuineness towards oneself o Theoretical knowledge o Practical knowledge o Intuitive knowledge o Reflective knowledge	
Cultural context with promoting and hindering norms		
Balancing act	A balancing act of the natural and medical perspectives o Exhibiting sensitivity for the genuine	
***The primacy of the good midwife in the midwifery services***	***A model of exemplary midwifery practice^b^***	***A woman-centred childbirth model^c^***
	P: Creates a setting that is respectful and reflects the woman’s needs/Supports the normalcy of birth Q & T: Calm/Belief in the normalcy of birth/Patience/Confidence/Clinical objectivity/Decisive	Limited physical comforting measuress→accommodative midwifery actionss→decision making
The midwife’s interpersonal competence	Q & T: Commitment to empowering women	Limited mutual participation and responsibility sharing→ participative decision making→ mutual participation and egalitarian relationship Limited information sharing and empowering→information sharing and empowering→equality with principle of power-sharing Ineffective communication→ interdependence and collaboration→partnership and collaboration
The midwife’s professional wisdom	P: Updates knowledge Q & T: Exceptional clinical skills/Judgement/Knowledge of self-limits	
The midwife’s professional competence The midwife’s professional caring	D: Caring O: The woman and family have a health care or birth experience that is respectful and empowering P: Respects the uniqueness of the woman & the family/Vigilance & attention to details Q & T: Commitment to the health of women & families/Humility/Gentle/ Realistic/Warmth/Understanding & supportive/Interest in others/Commitment to family-centred care	
The midwife’s development	D: Profession of Midwifery O: Enhancement of the Profession of Midwifery P: Balance of professional & personal life/Personal & peer review Q & T: Maturity/Wisdom/Persistence/Positive outlook/Commitment to the profession/ Accountability/Love of the work of midwifery/ Intelligence & intellectual curiosity/Possesses integrity &honesty/Motivation/Tolerance/Non-Judgmental/ Compassion	
	D: Therapeutics O: Optimal health of the woman and/or infant in the given situation	
		Responsibility sharing→Independence/Well informed to enhance self-esteem, self-determination and self-reliance
		Limited human and material infrastructure→maximising human and material infrastructure

a Components formed as three pairs representing women and midwives respectively.

b Components distinguish between Dimensions (D) with preceding Outcomes (O), Processes to achieve outcomes (P), and midwives’ Qualities and Traits (Q & T).

c Components are given as processes comprising: starting point→procedure→goal.

Below follows a summary of similarities and differences, with the reference model as a basis. We start with the components described in the reference model and end with the components not present in the reference model.

Birthing atmosphere: This component, or similar one, was evident in four models (Exemplary midwifery practice, Midwifery at high risk, Woman-centred SA, Woman-centred Nordic)^31-33,35^.

Reciprocal relationship: All six models broach a component about the relationship between woman and midwife in some way.

Grounded knowledge: This component, or similar meaning, exists in four models (Exemplary midwifery practice, Midwifery at high risk, The primacy of the good midwife, Woman-centred Nordic)^31,32,34,35^.

Cultural context: This component exists only in the reference model (Woman-centred Nordic)^35^.

Balancing act: This component, or similar one, was evident in four models (Exemplary midwifery practice, Midwifery at high risk, The primacy of the good midwife, Woman-centred Nordic)^31,32,34,35^. The midwife’s development and profession appeared in two models (Exemplary midwifery practice, The primacy of the good midwife)^31,34^.

Therapeutics: The goal of optimal health of the woman/infant was only part of one model (Exemplary midwifery practice)^31^.

Two processual concepts were evident in one model (Woman-centred SA)^33^: 1) the process of responsibility sharing, which leads to independence and enhanced self-reliance for the woman; and 2) human and material infrastructure. The mapping of the components revealed that only one model mentioned the family (Exemplary midwifery practice)^31^, and that the woman’s partner is not apparent as a part of any of the models.

## DISCUSSION

The aim of this study was to identify and obtain an overview of theoretical models for midwifery care. Below, we discuss our findings related to the characteristics of the included models, the scarcity of models and the underlying salutogenic perspective.

### Similarities and differences between the included models

We identified six models. There is variation in several characteristics among the models, for example the philosophical ideas underpinning the models, the methodology used to develop them, and the degree of complexity. Our conclusion was that all included models were generated with the intention to form an evidence-based theoretical basis for midwifery care, and none of the models had been developed based on earlier developed and published midwifery models of care. However, the mapping of the components revealed several differences, among them the content and extent of the models. Furthermore, the mapping of components revealed similarities, for example that all six models comprised a component relating to the relationship between the woman and the midwife. The analysis shows that the models are mainly oriented towards four dimensions: health, care, relationships and the midwifery profession.

The six defined studies originate from Sweden, Iceland, Scotland, the US, New Zealand and South Africa. The characteristics of the models might represent the context of the country from where they emerged, since the structure of healthcare differs, as well as the role and status of midwives^36^. In the Nordic countries, New Zealand and Scotland, midwifery exemplifies a profession that appears to be strong yet seems to be struggling to maintain independence. For example, the organization of maternity care appears fragmented in some of these countries, meaning that although women usually meet the same midwife throughout their pregnancy, they might be attended by an unknown midwife during birth and postpartum. Emphasis on relationships and midwifery knowledge is of importance to midwifery care in that context. In the US, although there is an intra-country difference, midwives generally provide only a small part of the care during pregnancy and birth^37^. This could be the reason for the thorough explanation of the model presented by Kennedy^31^, with emphasis on most of the components that are highlighted in the sample of models^38^. The South African model is developed in a tertiary hospital and highlights the woman in the center. It is a work built on the concept analysis of woman-centered care by the same author^39^. The model has its background in ‘Batho–Pele’, a political initiative in South Africa, and stands for better delivery of good service. This might explain the detailed description of the phases of the model’s development and the elements it outlines^33^.

The midwife-woman relationship was a common focus in all six models and two models specifically highlighted ‘woman-centered care’^33,35^. Woman-centered care has been referred to as a concept^40^, a tool, a framework, and a philosophy^41^. It has been associated with highquality maternity services and has been used to underpin organizational documents, and as a framework for policy documents and standards due to its strong midwifery-specific focus^40,41^. Woman-centered care has not been defined explicitly but is associated with a variety of care models and dimensions such as reciprocity, shared decision-making, continuity of care, relationship and empowerment^40,41^. Thus, the concept is closely linked to a midwife–woman relationship that is dynamic and reciprocal^41^. In a recent paper that develops a hierarchical model of the means and targets of midwifery, Peters et al.^42^ demonstrate that midwifery care is based on a trusting relationship. They further show that in order to establish a trusting relationship, midwives must provide individual and woman-centered care. Although only two of the models included in our paper specifically refer to the concept of woman-centered care^33,35^, all models refer to the dual relationship between the woman and the midwife. Furthermore, the models focus on supporting the woman’s autonomy and engaging her in the care process. These values are closely linked to woman-centered care, salutogenesis and a biopsychosocial model of childbirth^41^. Except for Kennedy’s^31^ model of exemplary midwifery practice, none of the models included in our review refers to the women’s baby, family or partner. According to Leap’s definition of the concept, woman-centered care includes the needs of the baby, family and other persons that are important to the woman ‘as defined and negotiated by the woman herself’^40^. She argues that, when women are empowered, they have the potential to empower their families and communities. Others argue that the scant referral to family, partner and child requires further attention and that these elements should be included in the theory^41,43^. Carolan and Hodnett^43^ even imply that the concept of woman-centered care in itself excludes the woman’s partner and her family.

### Scarcity of models

Only six studies were found to be eligible for inclusion. This small number could relate to the fact that midwifery, although having a very long history overall, only has a short history when it comes to developing knowledge and theory, and conducting research in the field of maternity care. Midwifery has been seen as a profession that does practical work. In Europe, the development of midwifery research was initiated in the English-speaking countries during the 1980s and 1990s^44^. As late as 2010, the term ‘midwifery’ was not a MESH or subject heading in many of the relevant databases^45^. This phenomenon could be one of the reasons why theoretical models based on systematic and scientific development are scarce, while descriptions of ways of organizing care are broad. This resonates with research developed in the aftermath of the development of a new evidence-informed quality maternal and newborn care (QMNC) framework^27^. A mapping of midwifery-led antenatal care models in relation to the QMNC framework showed that the organization of care was the best described component, while underlying values and philosophies concerning care were poorly reported^17^. There is reason to believe that the same phenomenon not only relates to models for antenatal care, but to all care models during the maternity episode.

### A salutogenic perspective

Several of the models seem to function from an underlying focus on what facilitates health rather than what hinders risks related to childbirth. This is expressed in the models’ goals and ideals which underline, for example, terms such as: normalcy of birth, presence, interpersonal competencies, and power-sharing or empowerment. These few examples enhance what could be interpreted as implicit also in medical models of maternity services, but are ostensibly not, for example the phrase supporting the normalcy of birth. Facilitating health is a more complex task than hindering demarcated risks, and somehow the models seem to reflect this task in their very depiction. All models are multi-directional, and attempt to incorporate the complexity in a care model, rather than focusing on risk-avoidance, which tends to be more one-directional^46^. This is also elaborated in several of the studies, for example in Kennedy^31^, who highlights ‘the art of doing nothing well’^31^. This is an expression comparable to what can also be found in the Lancet series on maternal health^47^, stressing that good quality maternity care should be ‘neither too much, too soon, nor too little, too late’^48^. Thus, there seems to be a tendency towards an underlying salutogenic focus cultivating the models^7^. This is in line with complexity theory, which challenges the behavior of a healthcare system as a linear process. The taxonomy for complexity theory has been developed to further understand how certain techniques and procedures become widely adopted^12^.

### Methodological considerations

The scope of this review was broad. We did not aim to provide an overview of organizational models of care that included models for providing care or services, but to map theoretical models for midwifery care that were developed in a scientific and systematic way. The literature searches were inclusive as there is no consensus on the meanings of the different terms used to describe models for midwifery care. Although we conducted extensive literature searches guided by an experienced librarian, our choice of search terms and inclusion criteria may have been inconclusive. The use of nine databases provided a comprehensive list of articles. We did not include grey literature or perform a manual journal search for additional papers, but we searched the reference lists of the papers included in the study.

Being a group of researchers residing in four different countries, we found computer software for managing the references helpful since it enabled us to work efficiently and simultaneously with the screening process. However, the software only allows for one screening prior to the full-text screening, and we found this challenging since our eligibility criteria were refined throughout the screening process. One of the authors (MB) is the first author of two of the included papers. We therefore arranged the screening process in a way that ensured that other members of the team made decisions about these two papers.

We are aware that our preunderstandings may have influenced our work. However, we conducted the analysis in collaboration and allowed time for discussion when we encountered concepts or parts of a model that were challenging to understand. This reduced the risk of selection bias.

Mapping reviews do not usually include a quality assessment process. Consequently, we did not assess or score the included papers, but we are aware that their quality differs to some extent.

## Conclusions

The aim of this study was to identify and gain an overview of theoretical models for midwifery care. Through the four key stages of the SALSA framework consisting of systematic searches, appraisal, synthesis and analysis, we identified six models originating from Sweden, Iceland, Scotland, the US, New Zealand and South Africa. Although stemming from different contexts, the included models seemed to share some characteristics, the most prominent being the relationship between the woman and the midwife, which was understood as an important component in all the models. This is interpreted as a shared grounded belief that midwifery care should be individual and woman-centered. Furthermore, we found a tendency towards an underlying salutogenic focus cultivating the models, which emphasizes health facilitation rather than risk hindering.

Overall, scarcity exists in relation to theoretical models for midwifery care with explicit epistemological status, contrary to the existence of many descriptions of ways of organizing care that are not epistemologically underpinned. This might be because of the recent and relatively short history of scientific theory-development and research in the field of midwifery care. Midwifery has been seen as a profession that does practical work. On the basis of our findings and analyses, we argue that a sound knowledge base needs to be theoretically based to be able to safeguard the midwifery profession and the underlying foci of, for example, women- and relation-centered approaches and a salutogenic point of departure. Therefore, there is a need for more research aimed at the development of theoretical models for midwifery care.

## CONFLICTS OF INTEREST

The authors have completed and submitted the ICMJE Form for Disclosure of Potential Conflicts of Interest and none was reported.
